# Associations between age, social reward processing and social anxiety symptoms

**DOI:** 10.1007/s12144-023-04551-y

**Published:** 2023-04-19

**Authors:** Emma J. Kilford, Lucy Foulkes, Sarah-Jayne Blakemore

**Affiliations:** 1 Institute of Cognitive Neuroscience, University College London, London, WC1N 3AZ UK; 2 Department of Clinical, Educational and Health Psychology, Division of Psychology and Language Sciences, University College London, Gower Street, London, WC1E 6BT UK; 3 Department of Experimental Psychology, University of Oxford, Oxford, OX2 6GG UK; 4 Department of Psychology, University of Cambridge, Cambridge, CB2 3EB UK

**Keywords:** Adolescence, Social reward, Social anxiety, Reward sensitivity, Reward processing, Social processing, Motivational processing

## Abstract

Reward processing undergoes marked changes in adolescence, with social interactions representing a powerful source of reward. Reward processing is also an important factor in the development of social anxiety disorder, a condition that most commonly first appears in adolescence. This study investigated the relationship between age, social reward processing and social anxiety in a cross-sectional sample of female participants (N = 80) aged 13–34. Participants performed two versions of a probabilistic reward anticipation task, in which a speeded response could result in different probabilities of receiving either social or monetary rewarding feedback. Participants also completed self-report assessments of social reward value, trait anxiety and social anxiety symptoms. At high reward probabilities, performance on both reward tasks showed a quadratic effect of age, with the fastest responses at around 22–24 years. A similar quadratic effect was found for subjective liking ratings of both reward stimuli, although these were not associated with performance. Social anxiety was not associated with a subjective liking of the rewards but did predict performance on both tasks at all reward probabilities. Age-related variation in reward processing was not accounted for by age-related variation in social anxiety symptoms, suggesting that, while both social anxiety and age were associated with variation in reward processing, their effects were largely independent. Together, these findings provide evidence that social reward processing continues to develop across adolescence and that individual differences in social anxiety should be considered when considering reward sensitivity during this period.

**Supplementary Information:**

The online version contains supplementary material available at 10.1007/s12144-023-04551-y.

## Introduction

Adolescence is the period of marked physical, social and behavioural change between the approximate ages of 10 to 24 years (Sawyer et al., 2018). This time of life is a period of vulnerability for mental health problems (Blakemore, 2019; Kessler et al., 2005), particularly social-emotional disorders (Rapee et al., 2019). This includes social anxiety disorder (SAD), which has been called ‘the prototypical adolescent disorder’ because it so often begins in the adolescent years: there is a marked increase in rates at age 10, and the median age of onset is 13 years. Approximately 75% of cases begin by age 15, and 90% by age 23 (Beesdo et al., 2010; Kessler et al., 2005; Stein, 2006).

### Social reward in adolescence

Reward sensitivity is the tendency to seek out, learn from, and experience pleasure from positive stimuli. This tendency undergoes marked changes in adolescence, with the majority of evidence suggesting that sensitivity to reward, such as money and food, is heightened in this period of life (van Duijvenvoorde et al., 2016). At the same time, adolescence is a period of social re-orientation, in which the social world and interactions with peers become particularly salient. For example, compared with children, adolescents form more complex and hierarchical peer relationships (Brown, 2004; Steinberg & Morris, 2001), and compared with adults, are more sensitive to acceptance and rejection by their peers (Sebastian et al., 2011; Somerville, 2013) and more concerned about taking social risks (Andrews et al., 2020b). Due to this parallel sensitivity to both rewards and social relationships, it has been proposed that adolescents might be especially sensitive to social rewards – such as viewing pictures of smiling faces, sharing with a friend, or being liked – and that this might affect their social behaviour (Andrews et al., 2020a; Foulkes & Blakemore, 2016).

Most evidence for heightened social reward sensitivity in adolescence comes from animal studies, whereas findings from human studies are less conclusive (Foulkes & Blakemore, 2016). Research with rodents suggests that social interactions may be more rewarding for adolescent animals than for adults, with adolescent rats showing a greater preference for social (social interaction) over non-social (amphetamine) rewards (Yates et al., 2013), and a more sustained dopaminergic release in response to social interactions (Robinson et al., 2011). In humans, behavioural studies indicate that images of smiling faces are more distracting for adolescents (12–14 years) than adults (18–29 years; Cromheeke & Mueller, 2015). Neuroimaging studies examining adolescents’ neural responses to social stimuli (e.g. happy face stimuli or being with one’s peers) have shown heightened activity in regions associated with reward processing, such as the ventral striatum (Chein et al., 2011; Somerville et al., 2011). However, this reverse inference is speculative and might not be valid as this region is also involved in processing salience (Levita et al., 2009). Therefore, it is unclear whether heightened activation in adolescence indicates greater reward value or greater salience of social stimuli at this age. For example, there is also evidence of activation of these regions in response to negatively valenced social stimuli in adolescence (Dreyfuss et al., 2014; Pfeifer et al., 2011), suggesting that adolescents might be hypersensitive to all social stimuli, not just social reward. Since few behavioural or neuroimaging studies have assessed the *subjective* value of socially rewarding stimuli (Foulkes & Blakemore, 2016), research is needed to examine whether changes in responses to social stimuli during adolescence result from a specific increase in the value of social rewards, or an increase in the salience of all social stimuli (both positive and negative) during this period of life.

### Social reward and social anxiety

Social reward processing has also been emphasised as an important factor in developmental models of SAD (Caouette & Guyer, 2014). SAD is defined as a persistent and impairing fear of one or more social or performance situations, in which an individual will be exposed to unfamiliar people and/or the possible evaluation or scrutiny of others (DSM-5; American Psychiatric Association, 2013). Individuals with SAD fear they will be embarrassed and humiliated by their behaviour or their anxiety symptoms, which means feared situations elicit intense arousal, distress and anxiety, or are avoided altogether. This then potentially creates a conflict, because adolescence is a period of social re-orientation, in which social stimuli and relationships are especially salient and potentially rewarding. Individuals with SAD may therefore experience a conflict between the desire to approach and avoid social situations: they are simultaneously highly invested in forming peers relationships and extremely fearful of humiliation or rejection (Caouette & Guyer, 2014; Lucock & Salkovskis, 1988).

This ‘approach-avoidance’ conflict could mean that individuals with SAD are less likely to seek out socially rewarding interactions, or that they are less likely to enjoy them when they happen. However, empirical evidence examining social reward processing in socially anxious individuals is limited when compared to the much wider body of research investigating fear and threat processing in SAD, and thus more research is needed if we are to be able to differentiate between these two possibilities. One fMRI study found that a group of adults with SAD showed decreased ventral striatum activation when anticipating social rewards (pictures of neutral faces) relative to controls, but no neural differences when receiving the rewards (Richey et al., 2014). The authors suggested this may be evidence that individuals with SAD show a reduction in approach-driven tendencies towards social stimuli (Richey et al., 2014). However, it should be noted that neutral faces may be perceived as threatening by socially anxious individuals (Cooney et al., 2006), complicating interpretation of the findings of Richey et al. (2014) and highlighting an important consideration for stimuli selection when assessing social reward (discussed further in Current study and research questions). A second study examining social reward (happy faces) and social punishment (angry faces) found that healthy controls showed heightened putamen activation for reward relative to punishment trials, which individuals with SAD did not (Cremers et al., 2015). In addition, individuals with SAD showed decreased putamen-anterior cingulate cortex connectivity relative to controls, for both reward and punishment trials. The authors interpreted these findings as evidence that, again, the typical motivation for social reward was attenuated in individuals with SAD (Cremers et al., 2015), but this was not supported by their behavioural data. For example, individuals with SAD did not have slower reaction times in the reward trials compared to controls. When asked to rate how much they subjectively liked the stimuli, they did not report liking the happy faces less than controls (Cremers et al., 2015). At a behavioural level, the relationship between social anxiety and social reward processing remains unclear.

### Current study and research questions

In the current study, we sought to understand the relationship between age, social anxiety and social reward in a community sample of girls and women, to capture a range of ages and levels of social anxiety. Symptoms of social anxiety exist on a continuum across the general population (Knappe et al., 2009). While the course of SAD, including age of onset, is similar across genders, girls and women are more likely to have SAD than boys and men, and in both clinical and community samples girls and women report greater prevalence and severity of symptoms (Asher et al., 2017; Caballo et al., 2014). These differences are most prominent during adolescence and decrease in magnitude across the lifespan (Asher et al, 2017; note that this paper reviews research examining both gender and sex differences in SAD, however the relative contributions of sex and gender influences on differences in prevalence, course and symptoms of SAD are not currently well understood).

We used a probabilistic reward task, in which participants respond to a cue that indicates a varying probability that a rewarding stimuli will be delivered, to assess the association between (1) age and (2) social anxiety symptoms on social reward processing. Reward probability was manipulated to provide a behavioural index of reward sensitivity, in line with previous studies showing that reaction times to reward cues can be modulated by varying either the magnitude/intensity of a reward or the likelihood of obtaining it (Demurie et al., 2012; Foulkes et al., 2014; Kahnt et al., 2014; Rademacher et al., 2010; Spreckelmeyer et al., 2009). The task had two reward conditions, social and monetary, and has been used previously as an index of reward sensitivity to both types of reward (Foulkes et al., 2014). We also assessed the subjective value of the reward symbols. In our paradigm, we included both social and monetary reward conditions as this allowed us to examine whether, if reward processing is associated with age and/or social anxiety symptoms, this the case for social reward processing specifically or due to a domain-general alterations in reward processing.

In some previous studies, monetary reward was represented with a currency symbol (e.g. a dollar sign) while social rewards were represented with images of smiling faces (Rademacher et al., 2010; Richey et al., 2014; Spreckelmeyer et al., 2009), which are more visually complex and biologically salient than a dollar sign. In the current study, we chose reward symbols to be as physically similar as possible, while representing different reward types. Social reward was represented by the ‘like’ symbol from the social networking site Facebook (www.facebook.com). This is a thumbs-up icon used to express approval/admiration – a well-established social reward (Foulkes et al., 2014; Rosenthal-von der Pütten et al., 2019) – from one user to another in response to items such as photos or comments. Note, that while Facebook usage among young people is currently declining in favour of other social media platforms (Anderson & Jiang, 2018; OfCom, 2022), at the time of this study over 80% of UK adolescents and young adults used Facebook (eMarketeer, 2013; OfCom, 2015). Monetary reward was represented by the pound sterling sign (£). Both symbols have a learnt association with reward, and both have simple visual features (Foulkes et al., 2014).

Using the like symbol, rather than smiling faces, has an additional advantage when examining social reward processing in relation to social anxiety. Social anxiety is associated with alterations in face processing, including an increased likelihood to interpret positive or neutral expressions as threatening (Cooney et al., 2006; Gilboa-Schechtman & Shachar-Lavie, 2013). Studies of socially anxious adults indicate automatic avoidance responses to both angry and smiling faces, despite explicitly rating the smiling faces positively (Rinck et al., 2013). As a result, when faces are used as a social reward it can be difficult to disentangle whether responses vary according to the reward value of the stimuli or the negative affective reactions these stimuli can elicit in socially anxious individuals. Furthermore, in some previous studies (particularly studies of adolescent development), social reward has been indexed by participants’ ability to ignore distracting social stimuli (Cohen-Gilbert & Thomas, 2013; Cromheeke & Mueller, 2015; Grose-Fifer et al., 2013; Hare et al., 2008; Somerville et al., 2011). However, ignoring these stimuli (if they are rewarding) requires inhibitory control. If a participant resists a stimulus, it is unclear whether this is due to finding it less rewarding or to having better inhibitory control ability. Therefore, the current task was designed to have no inhibitory control component.

This behavioural task was used in combination with questionnaire assessments to address the following research questions in a sample of female adolescent and adult participants:

1. **Is there age-related variation in the processing of social and monetary rewards during adolescence and early adulthood?** Based on studies suggesting that behavioural and neural assessments of reward sensitivity peak in the late teens and early 20 s (Braams et al., 2015; Urošević et al., 2012), it was predicted that age-related variation in reward sensitivity in both reward conditions (as assessed by subjective liking ratings and reaction times) would be characterised by a quadratic pattern. Given that few studies have assessed behavioural responses to social reward in adolescents and young adults in the context of other domains of reward (Foulkes & Blakemore, 2016), there were no directional predictions as to whether age-related variation in reward processing would differ according to reward type (social vs. monetary) or the probability of an upcoming reward.
2. **Is the processing of social and monetary reward associated with individual differences in social anxiety symptoms, and does this relate to age-related variation in reward processing?** Existing behavioural findings of social and reward processing in socially anxious individuals are mixed and it is currently unclear whether individuals with SAD are less likely to seek out socially rewarding interactions due to simultaneous heightened avoidance of potential social threats, or that they are less likely to enjoy them when they happen. Thus, we had no directional predictions regarding whether individual differences in social anxiety would be associated with variation in reward processing, or whether this would differ according to reward type (social vs. monetary) or the probability of an upcoming reward.

## Materials and methods

### Participants

80 female participants aged 13.12 to 34.53 years (*M* = 21.46, *SD* = 5.39) were recruited from university volunteer databases and schools in the Greater London area in 2015. There were no exclusion criteria, and the only inclusion criteria were that participants had to be current Facebook users and female (note that this was the language we used in recruitment adverts, and both were assessed by self-report). Only female participants were recruited due to the higher prevalence of SAD and symptoms observed in this population (Caballo et al., 2014) and as we did not have the resources to recruit a sample size large enough to control for or compare sex/gender and also have sufficient power to address our research questions.

The ethnicity of the sample was as follows: 58.8% Caucasian, 13.8% East Asian, 8.8% South Asian, 7.5% African/Caribbean, 7.5% mixed, 2.5% Latino, 1.3% not stated. The study was approved by the university ethics committee, and all participants, or their parent or guardian for those under the age of 18, gave written informed consent. Participants were tested individually, in either a quiet University laboratory room or a quiet room at their school. Participants who travelled to the University to take part were reimbursed £8 for their time plus travel expenses.

### Experimental reward task

The task was an adapted version of a probabilistic reward anticipation task first used in a previous study (Foulkes et al., 2014). As in Foulkes et al. (2014), two versions of the task (social and monetary reward) were used, with task order counterbalanced. Social reward was represented by the like symbol from Facebook, a thumbs-up icon used to express approval/admiration, whereas monetary reward was represented by the pound sterling symbol. For both task versions, the objective was to win as many points as possible. As in other studies that have compared monetary and social reward processing (Foulkes et al., 2014; Kohls et al., 2009; Rademacher et al., 2010), performance on the monetary task were not translated into actual monetary reward – to keep the two tasks as equivalent as possible. Participants who travelled to the University to take part were reimbursed £8 for their time, but this was not contingent on their performance. Instead, within the task we relied on the learned association between the two symbols and reward value.

**Fig. 1:**
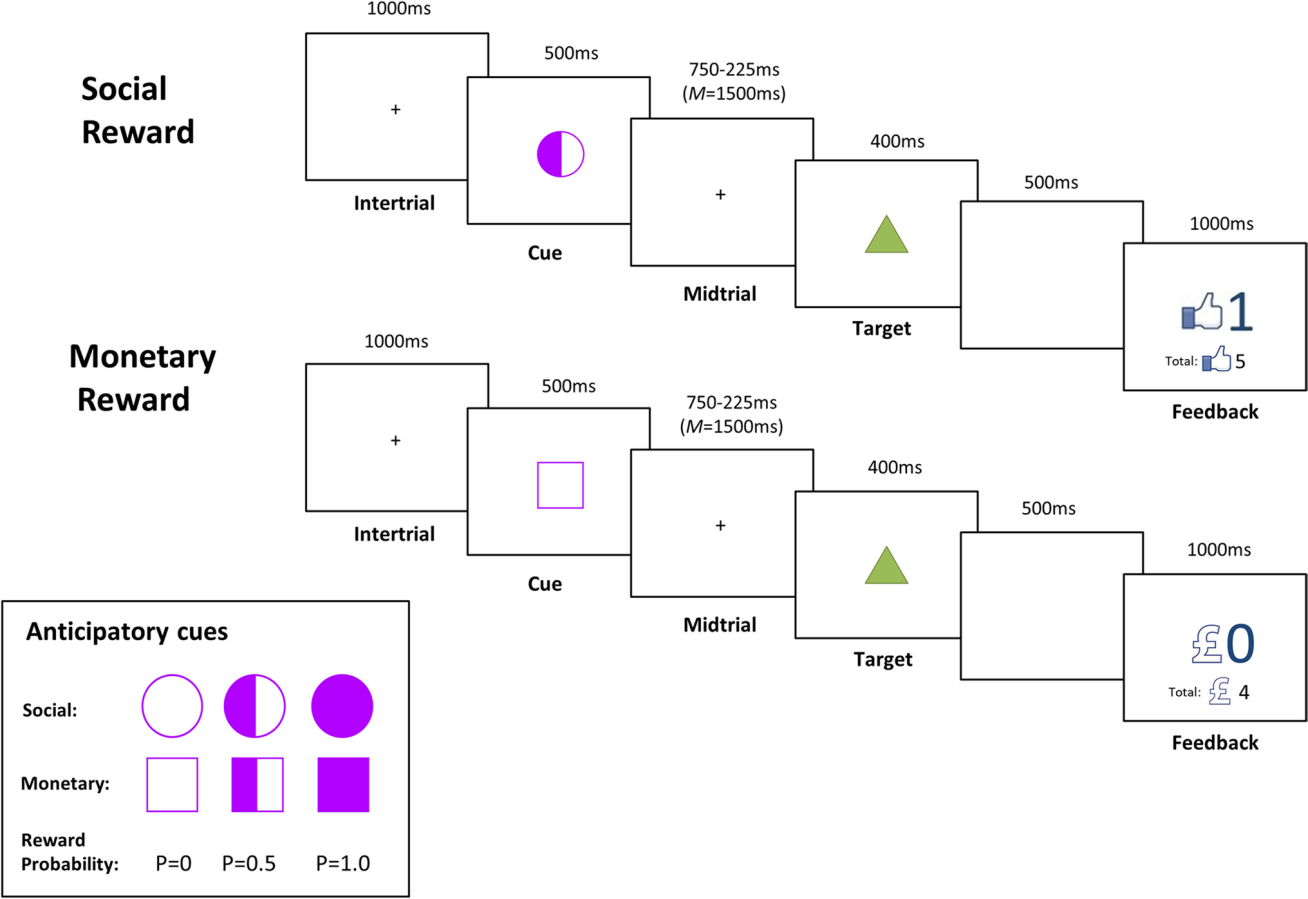
Trial sequence for the social and monetary reward tasks. In each task participants were required to respond to a triangular target with a button press as fast as possible. Before target presentation, participants saw one of three anticipatory cues (simple circle or square shapes) signalling the probability of receiving a reward, providing that the button was pressed fast enough (< 400 ms). Trial outcome was then presented on the feedback screen. Trials could result in either a reward outcome in the form of a point gain presented next to the reward symbol (either the Facebook ‘like’ or pound sterling symbol) or no-reward outcome (no point gain). *Adapted from *Foulkes et al. (2014)* with permission*

In both task versions, in each trial, the participant responded to a target (a simple green triangle) by pressing the space bar. They subsequently received feedback, which was either a reward (a social or monetary point gain) or no reward (no point gain; there is no loss condition). Before the target appeared, the participant saw one of three possible anticipatory cues (Fig. 1), which indicated the probability (P = 0, P = 0.5 or P = 1) that they would be rewarded if they responded quickly enough (within 400 ms) to the target on that trial. A response within 400 ms on a reward trial (i.e., all P = 1 trials and a randomised 50% of the P = 0.5 trials) resulted in a point gain, whereas on no-reward trials (all P = 0 trials and 50% of the P = 0.5 trials), participants did not receive a point, regardless of their response speed. Within each task version, the sequence of trials (P = 0, P = 0.5 or P = 1) was randomised for each participant. Participants subsequently saw a feedback screen, in which the outcome of the trial (1 or 0) was displayed next to the reward symbol (either the Facebook like or pound sign). Cumulative winnings for the task were also displayed underneath the trial winnings in order to maintain interest.

The timings and length of the task were adapted from the original paradigm (Foulkes et al., 2014) after piloting the task with adolescent and adult participants, in order to minimise boredom and fatigue effects and to shorten the task. Each trial therefore consisted of six sequential components with the following timings: (1) 1000 ms fixation cross/inter-trial interval, (2) 500 ms anticipatory cue, (3) 750–2250 ms (*M* = 1500 ms) fixation cross, (4) 400 ms green triangle target, (5) 500 ms blank screen, (6) 1000 ms feedback. Each trial lasted a total of 4.15–5.65 s. Each task contained 108 trials and lasted approximately 9 min. All participants completed a practice session of nine trials at the start of each task.

As in Foulkes et al. (2014), trials with reaction times (RTs) that were < 100 ms or > 900 ms (including any missing trials, i.e. those in which participants failed to respond at all) were considered invalid and excluded from analysis (2.1% of experimental trials: 1.1% social, 1.0% monetary). According to these criteria, no participant had > 20% invalid trials in either the social or monetary reward task and so data from all participants were included in the analysis. Mean RT for each probability level (0, 0.5 and 1) were calculated in both conditions (social and monetary) as indices of task performance for each participant, with faster RTs hypothesised to represent stimuli being more rewarding/salient (Demurie et al., 2012; Foulkes et al., 2014; Kahnt et al., 2014; Rademacher et al., 2010; Spreckelmeyer et al., 2009). Before using data from the task to assess our primary research questions (detailed in Data analysis procedure), additional analyses were performed as validation checks of the experimental paradigm (see SM.1 and SM.2, briefly summarised in Validation checks).

### Self-report assessments

#### Social anxiety

Participants’ social anxiety symptoms were assessed using the Liebowitz Social Anxiety Scale (LSAS; Liebowitz, 1987), which measures social anxiety in everyday life. Participants read a list of 24 social situations and report how much fear they feel in the situation (0 = None, 1 = Mild, 2 = Moderate, 3 = Severe) and how often they avoid it (0 = Never, 1 = Occasionally, 2 = Often, 3 = Usually). Items assess two core domains of social anxiety and SAD: general social interactions (11 items, e.g. ‘Meeting strangers’; *LSAS Social Interactions*) and performance situations (13 items, e.g. ‘Taking a test’; *LSAS Performance*). Subscale scores are calculated by summing the fear and avoidance scores within each subscale; both of these are summed for the total score. Participants in this study scored between 2 – 48 (*M* = 22.1, *SD* = 11.8) on *LSAS Social* *Interactions* (possible range: 0–66) and between 4 – 57 (*M* = 24.0, *SD* = 13.1) on *LSAS Performance* (possible range: 0–78).

#### Generalised anxiety

Generalised anxiety was assessed to differentiate the relationship between reward processing and broader anxiety symptoms, and reward processing and anxiety specific to the social domain. Generalised anxiety was measured using the trait subscale of the State-Trait Anxiety Inventory (STAI-T; referred to in this study as *STAI*; Spielberger, 1973). Participants read a list of 20 items (e.g., ‘I feel nervous and restless’; ‘I worry too much over something that doesn’t really matter’) and respond how often they ‘generally’ feel that way (1 = Almost never, 2 = Sometimes, 3 = Often; 4 = Almost always). The possible range of scores is 20 – 80, and participants in this study had *STAI* scores between 22 – 63 (*M* = 42.4, *SD* = 9.4).

#### Subjective Symbol Liking and Familiarity Ratings

After completing the reward tasks, participants rated how much they liked each reward symbol (Facebook like and pound sterling sign) using a visual analogue scale. This was a horizontal line on the computer screen, with no numbers or markers, which had ‘*Not at all*’ at one end and ‘*Very much*’ at the other. Using the mouse cursor, participants indicated where their answer fell on the line, and this was then recorded as a score between 0 and 30. (Participants did not see their recorded score for each scale, to minimise the chance that their answer in one condition would influence their answer in the other.)

Participants also rated how familiar they were with the reward symbols using the same scale. The reward task used here relies on the fact that participants will have a learned association between abstract symbols (Facebook like, pound sterling) and real rewards. The strength of this association likely varies according to symbol familiarity. Since experience tends to increase with age, symbol familiarity is likely to increase systematically with participant age. Therefore, to avoid confounding the extent to which participants liked the reward symbols with merely how familiar they were with them, symbol familiarity was entered as a covariate for all analyses of subjective liking ratings.

#### Social reward

Participants also completed the Social Reward Questionnaire for Adolescents (*SRQ-A*; Foulkes et al., 2017), a measure of individual differences in the value of five distinct domain of social reward: *Admiration* (being flattered and gaining attention)*, Negative Social Potency* (being cruel to others for personal gains)*, Passivity* (giving others control)*, Prosocial Interaction*s (treating others with kindness), and *Sociability* (engaging in group interactions; SM.2)*.* In the *SRQ-A*, participants are asked to what extent they agree with a series of statements (e.g., ‘I enjoy going to parties’; ‘I enjoy seeing others get hurt’). They give answers on a seven-point scale (0 = Strongly disagree, 7 = Strongly agree). Higher scores indicate that the participant finds that domain of social interaction more rewarding.

The *SRQ-A* was included as a second validation check of the experimental paradigm (in addition to the subjective liking ratings described above). Specifically, given that the Facebook like symbol represents social admiration and/or approval, it was hypothesised that performance on the social reward task (but not the monetary task) would be associated with participants’ self-reported enjoyment of receiving the approval of others, as measured by the *SRQ-A Admiration* subscale.

### Procedure

Participants performed the first session of the experimental reward task (either social or monetary; counterbalanced across participants) and then completed the matrix reasoning subscale of the Wechsler Abbreviated Scale of Intelligence (WASI; Wechsler, 1999). Due to concerns regarding the suitability of this subscale as an age-standardised assessment of non-verbal ability that emerged during analysis WASI performance was not analysed (see SM.3 for further details). Participants then performed the second session of the experimental reward task (approximately 5–10 min after the end of the first session) before rating their liking and familiarity of the two reward symbols. Questionnaire assessments of Social Reward (*SRQ-A*; Foulkes et al., 2017) and Social Anxiety (*LSAS*; Liebowitz, 1987) were completed either in advance of the session (30.0%) or, where participants failed to complete them in advance, at the end of the session (70.0%).

### Data analysis procedure

#### Is there age-related variation in the processing of social and monetary rewards?

Hierarchical (step-wise) linear regression models were used to investigate whether the value of different kinds of reward, as measured by (1) subjective liking ratings and (2) task performance (mean RT), varied with age*. *A priori power calculations using G ∗ Power 3.1.3 indicated that a sample size of 68 participants provided power of 0.80 to detect a medium effect size at a probability level of 0.05 (Faul et al., 2007).

##### Subjective liking

Symbol familiarity was entered first, to control for the effects of familiarity on liking ratings (*Step 1*). Linear (*Step 2*) and quadratic (*Step 3*) age regressors were then added in turn, with subjective liking ratings as the outcome variable. Improvements in model fit at each step were assessed by examining the significance of the *F* change. This model was run twice, once each for the social task and monetary task.

##### Reaction time

Six models were run, one for each probability level of the social and monetary reward conditions. For each model, to assess the extent to which associations between age and mean RT could be accounted for by age-related variation in subjective liking, liking ratings were included as the first step in the regression model (*Step 1*). Linear (*Step 2*) and quadratic (*Step 3*) age regressors were then added in turn and improvements in model fit at each step were assessed by examining the significance of the *F* change.

#### Is the processing of social and monetary reward associated with individual differences in social anxiety symptoms, and does this relate to age-related variation in reward processing?

To assess whether reward processing was influenced by individual differences in social anxiety, the two sets of hierarchical linear regression analyses used to assess age-related variation in (i) subjective liking ratings and (ii) mean RT were modified to include self-report measures of anxiety. In these analyses, *STAI* was always first controlled for, to try and enable differentiation between effects of social anxiety, and general anxiety, i.e. anxiety that is not necessarily specific to the social domain, on reward processing. Following that, the two *LSAS* subscales were then included in the same block, to enable examination of the unique effects of each subscale.

Where social anxiety was a predictor of reward processing, as assessed by i) liking ratings and ii) task performance (see Research question 2 for results of these initial analyses), a modified version of the hierarchical linear regression used to assess age effects (Is there age-related variation in the processing of social and monetary rewards?) was used to examine the extent to which variation in social anxiety accounted for age effects in reward sensitivity. *STAI* was included as the first step in the regression model (*Step 1*), and then the two *LSAS* subscales were included in the same block, to enable examination of the unique effects of each subscale (*Step 2*). Linear (*Step 3*) and quadratic (*Step 4*) age regressors were then added in turn and improvements in model fit at each step were assessed by examining the significance of the *F* change.

While it was not the main focus of this research question, for completeness we also used hierarchical linear regression models to assess the relationship between age and self-reported measures of social and general anxiety (see SM.4). Based on epidemiological findings that SAD rates of onset peak in early adolescence (Beesdo et al., 2010; Kessler et al., 2005; Stein, 2006), it was predicted that SAD symptoms would likely either decrease or remain stable with age.

## Results

### Validation checks

Before investigating our primary research questions, several preliminary analyses were performed as validation checks of the experimental paradigm to ensure that the symbols used in the task were serving as effective rewards. This analysis indicated that, as intended, participants responded significantly more quickly with each increase in reward probability (0 to 0.5; 0.5 to 1) in both social and monetary tasks (see SM.1 for full details). Based on subjective liking ratings, there was evidence that participants found the pound sign more likeable (*t*(79) = 5.1, *p* < 0.001) than the Facebook like symbol (Social: *M* = 18.1, *SD* = 8.0; Monetary: *M* = 22.8, *SD* = 6.9). There was no effect of reward type on symbol familiarity ratings (*p* = 0.263).

In a second validation check, we analysed the relationship between task performance (mean RT) and participants’ self-reported enjoyment of different types of social rewards (*SRQ-A* subscales) using correlational analyses (SM.2). The purpose of this analysis was to determine whether participants were differentiating between the two reward domains (social vs. monetary), as opposed to simply being influenced by the point gain in a domain-general manner, regardless of the specific nature of the reward symbol (Demurie et al., 2012). In line with predictions (see Social reward), *SRQ-A Admiration* was significantly negatively associated with RTs to social rewards in all three probability conditions, with those participants who reported greater enjoyment of being admired by others being faster to respond to targets in the social version of the reward task (Table S3). *SRQ-A Admiration* was not associated with RTs to monetary rewards, and other *SRQ-A* subscales were not associated with RTs in either task condition, suggesting that the social reward task was specifically sensitive to individual differences in the value of receiving approval/admiration from others, rather than simply reflecting a more domain-general variation in reward sensitivity (e.g. a drive to earn points).

### Research question 1: Are there age-related variation in the processing of social and monetary rewards?

#### Subjective liking

**Fig. 2:**
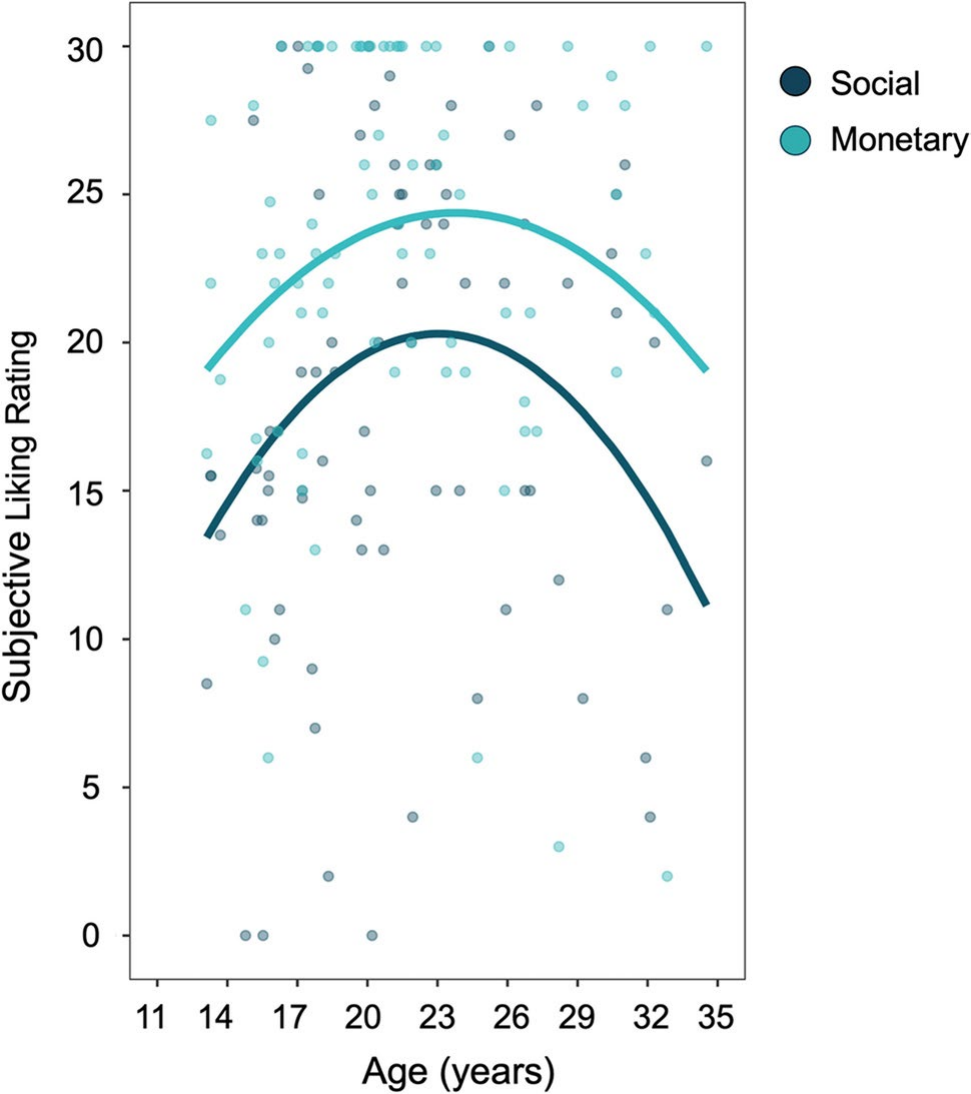
Age-related variation in subjective liking ratings of social and monetary rewards. Mean-standardised predicted values (lines) and raw data (dots) are plotted for subjective liking ratings of the reward symbols. Symbol familiarity ratings were first covaried, then the residuals were fitted by age^quadratic^ and plotted as a function of age

Hierarchical linear regression models were used to assess the relationship between self-reported liking of the task stimuli and participant age. Symbol familiarity was entered as a control variable (*Step 1*), followed by linear (*Step 2*) and quadratic (*Step 3*) age regressors, with subjective liking ratings as the outcome variable. Symbol familiarity was a significant predictor of subjective liking ratings for both the Facebook like and pound symbols (*p* < 0.001, see Table 1), accounting for 17.2% and 18.5% of variance, respectively. There was a significant quadratic effect of age on subjective liking ratings of both symbols (*Step 3*), which in combination with symbol familiarity accounted for 24.5% of variance in ratings of the Facebook like symbol (*p* = 0.008) and 23.1% of variance in ratings of the pound symbol (*p* = 0.042). Visual inspection of the data suggests that, for both reward types, subjective liking was highest around 23–24 years of age (quadratic effect plotted in Fig. 2). There was no linear effect of age (*Step 2*; *ps* > 0.586; see Table 1 for all fitted models).

**Table 1:** Effects of age on subjective liking ratings of the reward symbols

	Symbol Liking							
	Facebook like				Pound symbol			
	*R*^*2*^	*F*Δ	*pF*Δ	*β*	*R*^*2*^	*F*Δ	*pF*Δ	*β*
*Step 1*	0.172	16.20	< 0.001		0.185	17.67	< 0.001	
Familiarity				0.415***				0.430***
*Step 2*	0.172	0.00	0.981		0.188	0.30	0.586	
Familiarity				0.414***				0.431***
Age				0.003				0.056
*Step 3*	0.245	7.32	0.008		0.231	4.27	0.042	
Familiarity				0.377***				0.429***
Age				2.214**				1.738*
Age^2^				–2.219**				–1.694*

Summary of hierarchical regressions investigating linear (age) and quadratic (age^2^) effects of age on subjective liking ratings of social (Facebook like) and monetary (£) rewards. Symbol familiarity was controlled for as the first step of the model. *N* = *80.* ** *p* < 0.01, *** *p* < 0.001

#### Reaction times

Hierarchical linear regression analyses were used to assess the relationship between RT and participant age. Six models were run, one for each probability level for both social and monetary conditions. To assess whether any relationship between RT and age could be accounted for by age-related variance in participants’ subjective liking, liking ratings were included as the first step in the regression model (*Step 1*). Linear (*Step 2*) and quadratic (*Step 3*) age regressors were then added in turn, with RT as the outcome variable.

**Fig. 3:**
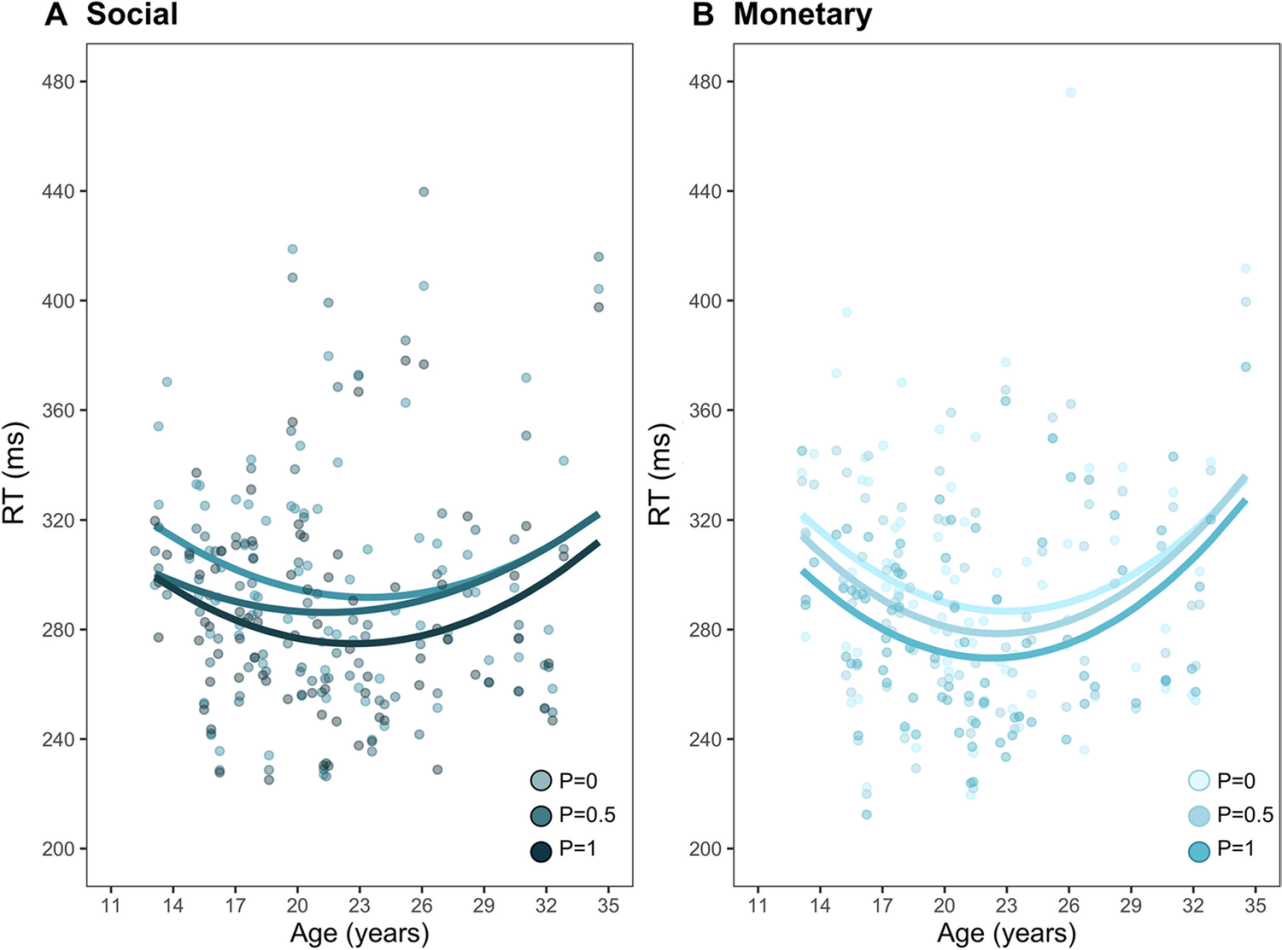
Age-related variation in social (**A**) and monetary (**B**) reward task performance (mean RT) at different reward probabilities. Mean-standardised predicted values (lines) and raw data (dots) for RT are plotted. Symbol liking ratings were first covaried, then the residuals were fitted by age^quadratic^ and plotted as a function of age

Subjective liking of the respective symbols did not significantly account for variance in RT at any probability level for either reward condition (*Step 1*; *p*s > 0.556), nor was there a linear effect of age on RT in any model (*Step 2*; *p*s > 0.413; Table 2). In the monetary reward condition, there was a quadratic effect of age at all probability levels (*Step 3; ps* < 0.026; Table 2). When P = 0, this accounted for 6.4% (*p* = 0.026) of variance in RTs; when P = 0.5, 11.7% of variance (*p* = 0.002); and when P = 1, 13.3% of variance (*p* = 0.002; see Fig. 3). In the social reward condition, there was a significant quadratic effect of age on RT when P = 1 (*Step 3*), accounting for around 5.8% of the variance (*p* = 0.037), but not when P = 0 or P = 0.5 (*p*s > 0.087; Table 2). Visual inspection of the data indicates that faster RTs to both types of reward occur around 22–24 years.

**Table 2:** Effects of age on social and monetary reward task performance (mean RT)

	Reward Probability											
	P = 0				P = 0.5				P = 1			
	*R*^*2*^	*F*Δ	*pF*Δ	*β*	*R*^*2*^	*F*Δ	*pF*Δ	*β*	*R*^*2*^	*F*Δ	*pF*Δ	*β*
**Social**												
*Step 1*	0.001	0.06	0.809		0.004	0.35	0.556		0.001	0.06	0.815	
Liking				0.027				0.067				0.027
*Step 2*	0.001	0.05	0.818		0.012	0.56	0.456		0.001	0.05	0.821	
Liking				0.030				0.058				0.024
Age				-0.026				0.085				0.026
*Step 3*	0.039	3.00	0.087		0.034	1.80	0.184		0.058	4.53	0.037	
Liking				0.097				0.110				0.105
Age				-1.700^+^				-1.211				-2.008*
Age^2^				1.680^+^				1.301				2.041*
**Monetary**												
*Step 1*	0.001	0.07	0.790		0.000	0.00	0.966		0.003	0.24	0.627	
Liking				0.030				-0.005				-0.055
*Step 2*	0.001	0.00	0.979		0.003	0.23	0.629		0.012	0.68	0.413	
Liking				0.030				-0.007				-0.059
Age				0.003				0.055				0.093
*Step 3*	0.064	5.13	0.026		0.117	9.83	0.002		0.133	10.66	0.002	
Liking				0.085				0.066				0.016
Age				-2.081*				-2.747**				-2.797**
Age^2^				2.098*				2.820**				2.909**

Summary of hierarchical regressions investigating linear (age) and quadratic (age^2^) effects of age on social and monetary reward task performance (mean RT) at different reward probabilities. Subjective liking ratings of the reward symbols were controlled for in the first step of the model. *N* = 80. ** *p* < 0.01, * *p* < 0.05, ^+^ *p* < 0.01

In addition, visual examination of the social reward task data (Fig. 3A) suggested that when P = 0.5 (i.e. when reward was uncertain, even when they responded quickly enough), younger participants responded similarly to when P = 1 (i.e. when reward was certain, provided they responded quickly enough). In contrast, older participants responded to P = 0.5 at a similar speed to when P = 0 (i.e. when there was no chance of reward, regardless of response speed). Thus, exploratory post hoc analyses were conducted to examine the possibility that responses to uncertain (P = 0.5) social rewards changed with age (see SM.5).

These exploratory analyses were consistent with visual examination of the data. Younger participants (< 20.5 years) showed significantly faster RTs when there was a possibility of a reward (P > 0) compared to when rewards were unobtainable (P = 0), but RTs did not significantly differ between uncertain and certain social reward trials (RT_P=0_ > RT_P=0.5_ = RT_P=1_; Table S5, Fig. S2). In contrast, older participants (> 20.5 years) did not differ between trials in which social reward was either unobtainable or uncertain (P < 1) but responded significantly faster when successful performance was certain to result in a social reward (RT_P=0_ = RT_P=0.5_ > RT_P=1_). This was not found for performance on the monetary reward task, where for all participants RTs were significantly faster when reward was certain (P = 1) than when it was unobtainable or uncertain (RT_P=0_ = RT_P=0.5_ > RT_P=1_).

### Research question 2: Is the processing of social and monetary reward associated with individual differences in social anxiety symptoms, and does this relate to age-related variation in reward processing?

#### Subjective liking

Hierarchical linear regression models were used to assess the relationship between social anxiety and subjective liking ratings of the reward symbols. Symbol familiarity was first entered as a control variable (*Step 1*), as it was a significant predictor of subjective liking ratings for both symbols (Research question 1; *p* < 0.001, see Table 1). General anxiety (*STAI)* was entered in the next block of the model *(Step 2*) to control for individual differences in anxiety that were not specific to social contexts, and then the two *LSAS* subscales were included in the following block, to enable examination of the unique effects of each subscale of social anxiety (*Step 3*).

Subjective liking ratings of each of the reward task stimuli were not significantly predicted by *STAI* (*Step 2; p*s > 0.640) or *LSAS* subscales (*Step 3; ps* > 0.523; SM.6, Table S6). Since liking ratings were not predicted by social anxiety, it was not necessary to investigate the role of social anxiety in age-related variation in reward value (assessed by subjective liking ratings), and thus this model was not extended to include age regressors.

#### Reaction times

Hierarchical linear regression models were used to assess the relationship between social anxiety and RTs. As in the age analyses (Research question 1), six models were run, one for each probability level of the social and monetary reward conditions. As subjective liking ratings were not associated with RTs at any probability level or in either reward condition (*p*s > 0.556, see Research question 1), here this step was dropped from the model for simplicity. Therefore, for each model, general anxiety (*STAI*) was entered first as a control variable (*Step* 1) to control for individual differences in anxiety that were not specific to social contexts, and then the two *LSAS* subscales were entered together in the following block, to enable examination of the unique effects of each subscale (*Step 2*).

**Fig. 4:**
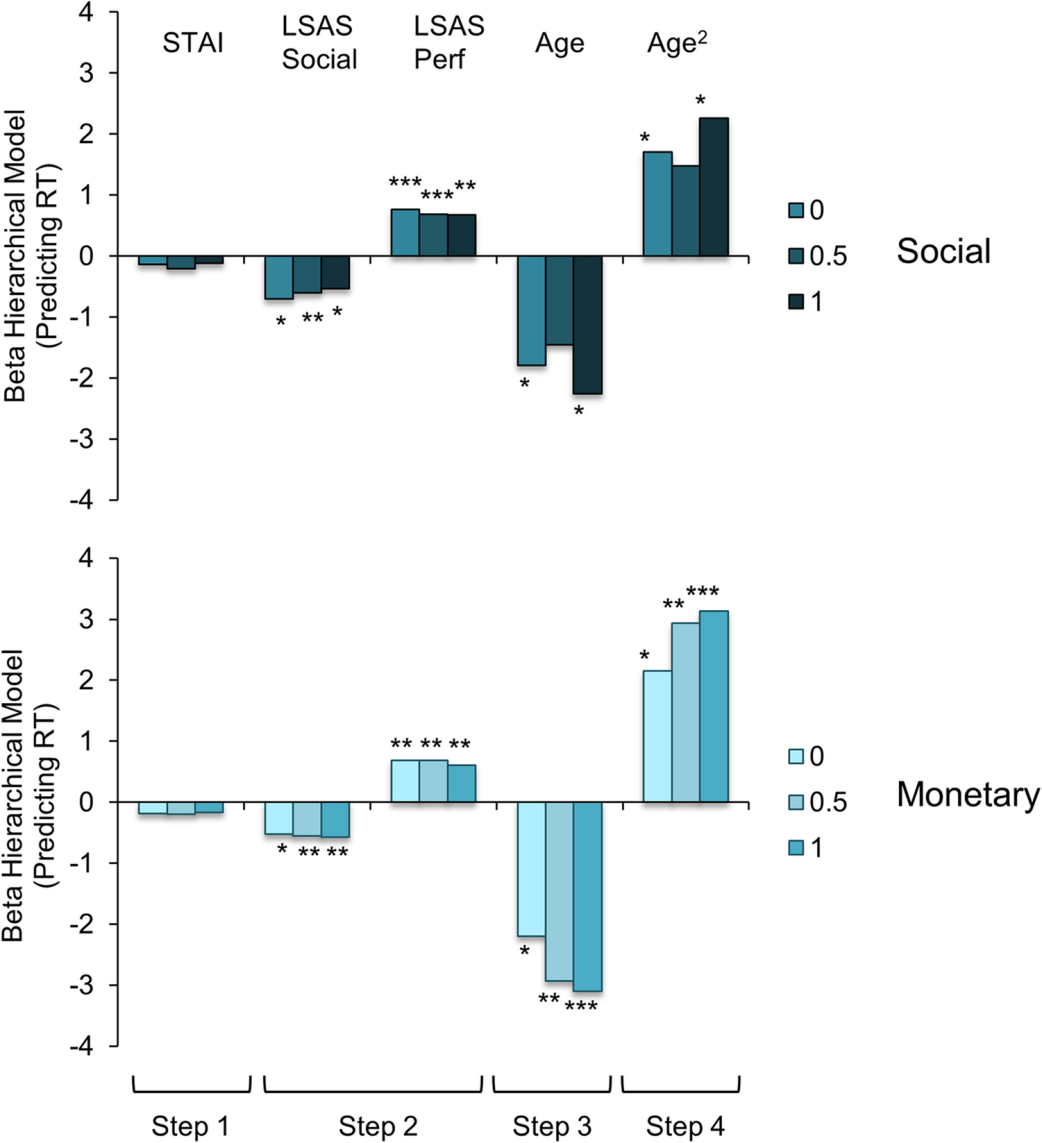
Effects of social anxiety and age on social and monetary reward task performance (mean RT). Visual summary of hierarchical regression analyses presented in Table 3. General anxiety (*STAI*), social anxiety symptoms (*LSAS Social Interactions* and *Performance* subscales) and linear (age) and quadratic (age^2^) effects of age on RTs in the social and monetary reward conditions across different reward probabilities were entered into a hierarchical regression model. The four steps correspond to the order in which the variables were entered. Betas from *Step 4* of the hierarchical regression models depicted in Table 3 are plotted. *** *p* < 0.001, ** *p* < 0.01, * *p* < 0.05

General trait anxiety *(STAI; Step 1)* did not significantly predict RTs in either reward condition, at any level of reward probability (*p*s > 0.091, Table 3). However, inclusion of the two *LSAS* subscales (*Step 2*) resulted in an improvement in model fit, accounting for an additional 8.4 – 13.4% of the variance in RTs (*p*s < 0.032; Table 3). Across reward probabilities, in both reward conditions, *LSAS Social Interactions* was a significant negative predictor of RTs, while *LSAS Performance* was a significant positive predictor (see Table 3). In other words, after controlling for general trait anxiety, participants with higher levels of social anxiety about social interactions showed faster RTs on the reward tasks, and participants with higher levels of social anxiety about performance situations specifically showed slowed RTs on the reward tasks (Fig. 4).

**Table 3:** Effects of social anxiety and age on social and monetary reward task performance (mean RT)

	Reward Probability											
	P = 0				P = 0.5				P = 1			
	*R*^*2*^	*F*Δ	*pF*Δ	*β*	*R*^*2*^	*F*Δ	*pF*Δ	*β*	*R*^*2*^	*F*Δ	*pF*Δ	*β*
**Social**												
*Step 1*	0.012	0.94	0.355		0.036	2.93	0.091		0.004	0.31	0.582	
STAI				-0.109				0.190^+^				-0.063
*Step 2*	0.146	5.97	0.004		0.154	5.26	0.007		0.114	4.73	0.012	
STAI				-0.121				-0.207^+^				-0.114
LSAS-S				-0.629**				-0.580**				-0.487*
LSAS-P				0.716**				0.670**				0.644**
*Step 3*	0.156	0.84	0.361		0.154	0.00	0.950		0.115	0.02	0.877	
STAI				-0.138				-0.205				-0.116
LSAS-S				-0.655**				-0.577*				-0.494*
LSAS-P				0.738**				0.668**				0.648**
Age				-0.102				0.007				-0.018
*Step 4*	0.199	3.90	0.050		0.178	2.19	0.143		0.172	5.09	0.027	
STAI				-0.142				-0.209^+^				-0.122
LSAS-S				-0.702*				-0.605**				-0.536*
LSAS-P				0.764***				0.688**				0.678**
Age				-1.798*				-1.459				-2.258*
Age^2^				1.703*				1.479				2.256*
**Monetary**												
*Step 1*	0.011	0.88	0.354		0.018	1.44	0.233		0.025	1.89	0.424	
STAI				-0.105				-0.135				-0.158
*Step 2*	0.125	4.93	0.010		0.127	4.72	0.012		0.109	3.59	0.032	
STAI				-0.171				-0.182				-0.159
LSAS-S				-0.460*				-0.491*				-0.513*
LSAS-P				0.645**				0.640**				0.565**
*Step 3*	0.127	0.21	0.651		0.127	0.01	0.943		0.109	0.03	0.881	
STAI				-0.179				-0.184				-0.156
LSAS-S				-0.478*				-0.494*				-0.507*
LSAS-P				0.656**				0.642**				0.561*
Age				-0.051				-0.008				0.018
*Step 4*	0.196	6.37	0.014		0.255	12.73	0.001		0.255	14.50	< 0.001	
STAI				-0.185				-0.192				-0.165
LSAS-S				-0.525*				-0.558**				-0.575**
LSAS-P				0.689**				0.687**				0.609**
Age				-2.200*				-2.931**				-3.100***
Age^2^				2.157*				2.935**				3.131***

Summary of hierarchical regressions investigating the relationship between social anxiety and age on social and monetary reward task performance (mean RT) at different reward probabilities. After controlling for trait anxiety (*STAI*; *Step 1*)*,* social anxiety symptoms (*LSAS Social Interactions* and *Performance* subscales) were entered into the model (*Step 2*). In *Steps 3* and *4* linear (age) and quadratic (age^2^) regressors were added to the model in turn. *N* = 80. LSAS*-S: LSAS Social Interactions;* LSAS*-P: LSAS Performance*; *** *p* < 0.001, ** *p* < 0.01, ** *p* < 0.05, + *p* < 0.1

An initial analysis of the relationship between age and self-reported measures of social and general anxiety indicated that age was significantly negatively associated with both *LSAS Social Interactions* and *STAI* scores (there was no significant association between age and *LSAS Performance* scores; see SM.4). This was generally consistent with our prediction that SAD symptoms would likely either decrease or remain stable with age and thus, where social anxiety was a significant predictor of RT, linear (*Step 3*) and quadratic (*Step 4*) age regressors were then added to the model in turn. This allowed us to examine the extent to which variation in social anxiety may have accounted for age-related variation in reward sensitivity on the task described in Research question 1. When age regressors were added to these models (Table 3, *Steps 3* and *4*), the effects of the two *LSAS* regressors on RT were retained or strengthened, as were all effects of age on RT, found in the models without the anxiety variables (see Table 2).

## Discussion

This study assessed whether sensitivity to social and monetary rewards varied with age and social anxiety, as measured in two ways: subjective liking of reward stimuli and RTs in a probabilistic reward task (with social and monetary conditions). For social and monetary rewards, both liking ratings and RTs were best characterised by a quadratic function of age, with greatest liking and fastest RTs peaking at around 22–24 years of age. This suggests that late adolescence/early adulthood may be a time of heightened sensitivity to social reward, in part because it is a time of heightened sensitivity to rewards more generally. Social anxiety symptoms were associated with RTs on both reward tasks, at all probability levels, with different domains of social anxiety showing opposing effects on RTs. This indicates a complex relationship between social anxiety and social reward processing. Individual differences in social anxiety did not account for the observed age-related variation in subjective liking ratings or RTs, suggesting these effects were largely independent from one and other.

Our findings of a peak in reward sensitivity in late adolescence/early adulthood is consistent with previous behavioural and neuroimaging studies that have found a quadratic pattern of developmental effects. For example, an fMRI study found that activation in the nucleus accumbens in response to monetary reward peaked around the age of 17 years (Braams et al., 2015); a behavioural study found that aspects of reward processing, primarily the tendency to approach rewards, peaked around 19 years (Steinberg et al., 2017); another found that scores on the Behavioural Activation Scale, a measure of general reward responsiveness, peaked in the late teens and then declined across the mid 20 s (Urošević et al., 2012). One of the few studies to assess age-related variation in social reward processing, as indexed by the self-report *SRQ-A*, found that the reward value of social approval peaked in the early 20 s (Altikulaç et al., 2019). The results of the current study therefore contributes to existing evidence that reward processing continues to develop into late adolescence and early adulthood, for both social and monetary rewards.

With regards to the question as to whether or not adolescence is a time of heightened sensitivity to social rewards, while the findings of the current study did indicate an increase in sensitivity to rewards of a social nature in late adolescence/early adulthood, they did not suggest that this was specific to social rewards, as similar increases were observed for monetary rewards. However, it should be noted that social reward is a complex and multi-dimensional construct, and therefore findings will vary considerably according to the specific paradigm used and the way in which social reward is operationalised. The social reward used in this study was associated with enjoyment of being admired but not with other aspects of social reward (Validation checks, SM.2). Thus, it may be that paradigms focussing on other dimensions of social reward may find different patterns of age-related effects. Furthermore, compared with other behavioural tasks assessing social reward processing in adolescence, our paradigm had relatively low cognitive and affective demands. Many studies have used non-abstract, socio-affective stimuli such as faces as social rewards (e.g. Cohen-Gilbert & Thomas, 2013; Cromheeke & Mueller, 2015; Grose-Fifer et al., 2013; Hare et al., 2008; Somerville et al., 2011), and have not included a non-social reward condition. While these studies have indeed found that such stimuli are more distracting for adolescents compared to adults, with such paradigms it can be difficult to disentangle developmental changes in (social) reward sensitivity from concurrent developmental changes in affective reactivity and cognitive control.

In our study, some surprising findings emerged with regard to uncertain social reward. In the monetary condition, RTs were characterised by a quadratic effect of age, across all probability levels. In contrast, in the social reward condition, RTs followed a quadratic effect only when P = 1 (i.e. when a fast response was certain to result in reward), and there was a trend toward a similar effect when there was no chance of reward (P = 0). When reward likelihood was uncertain (P = 0.5), age effects did not follow a quadratic effect. This indicates that response to uncertain social rewards may vary with age, which we investigated further with exploratory post-hoc analyses (SM.5).

This found that younger participants (< 20.5 years) showed similarly enhanced RTs to both certain (P = 1) and uncertain (P = 0.5) social rewards relative to the non-rewarded (P = 0) trials, whereas older participants (> 20.5 years) only showed faster responses when performance was certain to result in reward (P = 1). Despite being exploratory in nature, this finding yields interesting questions for future research. Uncertainty is an inherent property of real-world social rewards: we often know how much money we will earn in advance of engaging in a certain task or behaviour, whereas the extent to which our behaviour is likely to receive a social reward such as approval or admiration is much harder to predict. Younger adolescents are going through a period of extensive social development and establishing their place within peer networks; it might be that, at this younger age, the prospect of approval from an uncertain interaction is especially motivating (Andrews et al., 2020a; Nelson et al., 2005, 2016). Future studies would benefit from manipulating both magnitude and likelihood of social rewards to examine reward sensitivity across development.

Our second key finding was that RTs to social and monetary rewards varied as a function of individual differences in social anxiety, over and above variation in general trait anxiety (which was not associated with RTs). The fact that there were effects of social anxiety on RTs in both reward conditions suggests that alterations in reward processing in socially anxious individuals may not be specific to social rewards, consistent with findings of altered neural processing of monetary rewards in adolescents with, or at risk of, SAD (Guyer et al., 2006, 2012). In both the social and monetary reward conditions, anxiety specifically relating to social interactions (*LSAS Social Interactions*) was associated with faster RTs to reward stimuli at all reward probabilities, whereas anxiety specifically relating to performing/being observed was associated with slower responses (*LSAS Performance*).

Our finding of opposing directions of effects on RTs for these two facets of social anxiety could speculatively be understood within the framework of a performance monitoring hypothesis of reward processing in socially anxious individuals (Caouette & Guyer, 2014). This framework argues that the pattern of elevated striatal reactivity seen in socially anxious individuals in response to social or monetary gains and losses (e.g. Bar-haim et al., 2009; Guyer et al., 2006, 2012, 2014) reflects an increase in the salience of performance-contingent outcomes, resulting from a strong motivation to avoid failure or making errors, rather than reflecting differences in reward sensitivity per se (Lago et al., 2017). It has been suggested that similar to extrinsic rewards (e.g. money, admiration), intrinsic rewards (e.g. the inner drive to perform well) have an inverted U-shaped influence on task performance (van Duijvenvoorde et al., 2016), whereby as the salience of a performance-contingent outcome increases, performance improves up until a given point, at which the focus on the outcome becomes too great and hinders performance. In our study, although it was not a deliberate experimental manipulation, all participants performed the reward tasks in the presence of the experimenter, a social context which may have increased the salience of performance-contingent rewards for individuals with higher levels of social anxiety. While this could potentially serve to enhance performance to a degree in individuals with social anxiety experienced in social interactional situations, for individuals with high anxiety specifically regarding performance situations, the drive to perform well could have heightened outcome salience to the extent that it also impaired performance (i.e. slowed RTs). Although this suggestion is speculative, future research examining the effects of social anxiety on behavioural tasks could benefit from taking into account that the presence of an experimenter may have greater effects on performance in socially anxious individuals than non-anxious participants, and that the nature of these effects may differ according to symptom domains or severity, hypotheses that warrant further investigation.

In contrast to the RT data, social anxiety symptoms were not associated with subjective liking of either the social or monetary reward stimuli, a finding which is consistent with previous studies of socially anxious adults (Cremers et al., 2015; Heuer et al., 2007; Richey et al., 2014). For example, in a study that found socially anxious adults avoided both smiling and angry faces in a behavioural task, they did not differ from non-anxious controls in their ratings of the pleasantness of the smiling face stimuli (Heuer et al., 2007). This raises the possibility that although adolescents with high levels of social anxiety might find it more difficult to approach social stimuli or engage in social interactions, they nonetheless may still find them pleasurable when they do occur, which may have implications for understanding how best to support socially anxious adolescents to develop and maintain social relationships that they might find difficult but still potentially highly rewarding. A recent ecological momentary assessment study in which adults with SAD reported experiencing pleasure during naturally occurring social interactions, despite feelings of anxiety, and found socialising more pleasurable than being alone, suggests this may be the case for at least some socially anxious adults (Goodman et al., 2021).

Our final finding of note is that, although age-related variation in self-reported anxiety symptoms was observed, this did not account for the age-related variation observed in subjective liking ratings or RTs. Rather than diminishing the effects of age observed on RTs on the reward task, inclusion of social anxiety symptoms as predictors in the model increased the strength of age effects. This suggests that social anxiety and age both influenced performance on the reward tasks, but that that these influences were largely independent from one another.

It should be noted that our study relied on the learned association between the two symbols with rewards to reinforce behaviour, as opposed to using actual social and monetary rewards. This decision was made to keep the two conditions as equivalent as possible, an approach which has been used in other studies comparing the two types of reward (Foulkes et al., 2014; Spreckelmeyer et al., 2009; Rademacher et al., 2010). Participants were told that the objective of the reward tasks was simply to earn as many points as possible, with the reward being symbols that had learned associations with each type of reward. While the use of incentives undoubtedly heightens the salience of rewards, points and feedback images (sometimes referred to as cognitive incentives; Kray et al., 2018), are frequently used as incentives in the gamification of tasks and other non-gaming contexts (Richter et al., 2015; Alsaad & Durugbo, 2021) and can be effective behavioural reinforcers (e.g. Demurie et al., 2012).

To assess the validity of our experimental task as an index of sensitivity to social and monetary rewards, we conducted a series of validation checks (see Validation checks and SM.1 and SM.2). These indicated that, despite the fact that no actual reward was awarded on the basis of task performance, participants were sensitive to the differences in reward probability, both the social and monetary reward symbols were serving as effective behavioural reinforcers and that participants were differentiating between the two reward domains (social vs. monetary), as opposed to simply being influenced by the point gain in a domain-general manner. Yet, in the same way that using actual monetary reward enhances the ecological validity of a reward paradigm, studies using real or simulated social rewards have the potential to address important outstanding questions.

Working with individuals’ real-life social media content poses ethical challenges due to the sensitivity of this data, particularly in young people. However, paradigms that experimentally manipulate social media feedback in simulated online peer interactions have also been developed (Dziura et al., 2022; Sherman et al., 2016). Studies using such methods suggest that they are an effective method of manipulating social reward and can be sensitive to individual differences in the effects of online social interaction on mood. Sherman et al. (2016) found that when participants (aged 13–18 years) believed they were viewing their own photos on Instagram with liking ratings from peers, significantly greater neural activity was observed in multiple brain regions, including areas often implicated in social cognition and reward learning and motivation. Dziura et al. (2022) found that young people (aged 10–17 years) with a greater neural response to social rewards (simulated positive peer engagement) were more sensitive to the frequency of social interactions during the COVID-19 pandemic. This sensitivity was associated in both positive and negative effects depending on the nature of the type of social interaction engaged in. Future research using these controlled social environments has the potential to be able address important questions regarding the relationships between social reward and social punishment and/or the loss of a desired social reward within the same scenario and individuals.

One limitation of this study is the use of a non-clinical sample, limiting the generalisability and clinical application of our findings regarding social anxiety. However, affective disorders can also be considered from a continuous perspective, whereby behaviour varies across a continuum ranging from healthy to psychopathological (Kashdan, 2007). Nonetheless, future research should assess whether the current findings also apply to individuals with clinically-significant levels of social anxiety. Our finding that different domains of social anxiety symptoms had opposing influences on task performance (mean RT) suggests that there may be some utility in examining associations between specific symptoms of social anxiety and behaviour.

Another limitation of this study is that it only included female participants, in order to ensure power was not lost in a relatively small sample by needing to control for or compare sex and/or gender. Thus, we were unable to assess the influence of these factors on reward processing and social anxiety in our study. There is a higher prevalence of SAD and social anxiety symptoms in women (Caballo et al., 2014) and a study of healthy adults suggested there may be differences between men and women in electrocortical responses to social and monetary rewards (Distefano et al., 2018), thus our findings may only be generalisable to female samples. It should be noted that when this data was collected in 2015 we did not clearly establish whether we were capturing information about sex and/or gender when we advertised for ‘female’ participants. This limitation applies to many previous studies of social processing and anxiety, including epidemiological research describing the prevalence, course and symptoms of SAD. Given the that these two constructs are often strongly correlated, further research in which these factors are more clearly delineated will be needed if we are to try and understand the relative influences of sex and gender on SAD. We recommend that future studies consider the complexity of the relationships between these factors carefully at the point of research design and data collection (Clayton & Tannenbaum, 2016).

Lastly, future studies should assess a wider age range of participants. Due to the fact that we only included Facebook users in this study, and that all participants we approached under the age of 13 did not use Facebook (13 is the minimum age required to create a Facebook account), we were unable to include a younger age range within this study design. However, in order to fully examine developmental trajectories of social and non-social reward processing, studies should include children as well as adolescents and adults.

## Conclusions

In the current study we demonstrated that for both social and monetary rewards, subjective liking ratings and reaction times on behavioural reward processing task showed quadratic effects of age, peaking in the early twenties. Social anxiety was not associated with variation in subjective liking ratings of either the social or monetary reward stimuli but did predict RTs on both reward tasks at all probability levels. Together, these findings support evidence that reward processing continues to develop during late adolescence and into young adulthood. While changes in reward and socio-affective processing may contribute to heightened social concerns during adolescence, individual differences in social anxiety should be taken into account when considering sensitivity to rewards throughout adolescence and into adulthood.

## Supplementary Information

Below is the link to the electronic supplementary material.

Supplementary file1 (PDF 345 KB)

## Data Availability

We cannot share the data for ethical reasons, because we did not ask participants for permission to make their data publicly available at the time of collection.
