# Supplementary material for: Associations between age, social reward processing and social anxiety symptoms
Source: Curr Psychol. 2023 Apr 19;43(5):4305–22. doi: 10.1007/s12144-023-04551-y (PMC10113964; doi:10.1007/s12144-023-04551-y)
Supplement: Supplementary file 1 — Supplementary file1 (PDF 345 KB) [file 12144_2023_4551_MOESM1_ESM.pdf]

Supplementary Materials

SM.1 Validation of experimental paradigm (1)

As in Foulkes et al. (2014), reaction time (RT) was used as an index of reward sensitivity, with faster speeds hypothesised to represent a greater motivation to obtain the stimuli. This is in line with other studies of reward processing that have found stepwise effects of reward magnitude/intensity on RTs for both social and monetary rewards (Demurie et al., 2012; Sprecklemeyer et al., 2009; Rademacher et al., 2010). In Foulkes et al. (2014), initial behavioural analyses indicated a stepwise increase in RTs as reward probability increased for both social and monetary conditions, demonstrating that (1) participants were sensitive to the differences in reward probabilities and (2) both the social and monetary reward symbols were serving as reinforcers. These analyses were therefore repeated as a validation check to ensure that this was also the case within our younger sample.

Mean RTs were analysed using a 2 (reward type: social, monetary) × 3 (reward probability: 0, 0.5, 1) ANOVA. There was a significant main effect of reward probability (F (1.86, 147.52) = 40.81, $p < .001$, $\eta^2_p = .341$), but no main effect of reward type ($p = .472$) and no interaction between reward type and reward probability ($p = .995$). Participants responded more quickly as reward probability increased in both social and monetary tasks (**Fig. S1; Table S1**). Pairwise comparisons (Bonferroni corrected) showed that the decrease in RT between both increases in reward probability (0 and 0.5; 0.5 and 1) were significant in both social and monetary conditions (**Table S1**). These findings replicate those of Foulkes et al. (2014), thus indicating that both reward stimuli were capable of reinforcing behaviour in line with the expected likelihood of obtaining the reward.

Table S1. Social and monetary reward task performance (mean RT)

			Pairwise Comparisons	
**Reward**	**Probability**	**Mean RT *(SE)***	**(*A-B*)**	***A* - *B* (SE)**
	0	299.80 (4.61)	0 – 0.5	**7.57 (2.65) ***
**Social**	0.5	292.23 (4.92)	0 – 1	**16.91 (2.53) *****
	1	282.89 (4.05)	0.5 – 1	**9.34 (2.62) ****
	0	297.93 (5.05)	0 – 0.5	**7.42 (2.47) ***
**Monetary**	0.5	290.51 (4.17)	0 – 1	**17.07 (2.82) *****
	1	280.85 (3.68)	0.5 – 1	**9.66 (1.82) *****

Corrected p values are shown. *** $p < .001$, ** $p < .01$, * $p < .05$.

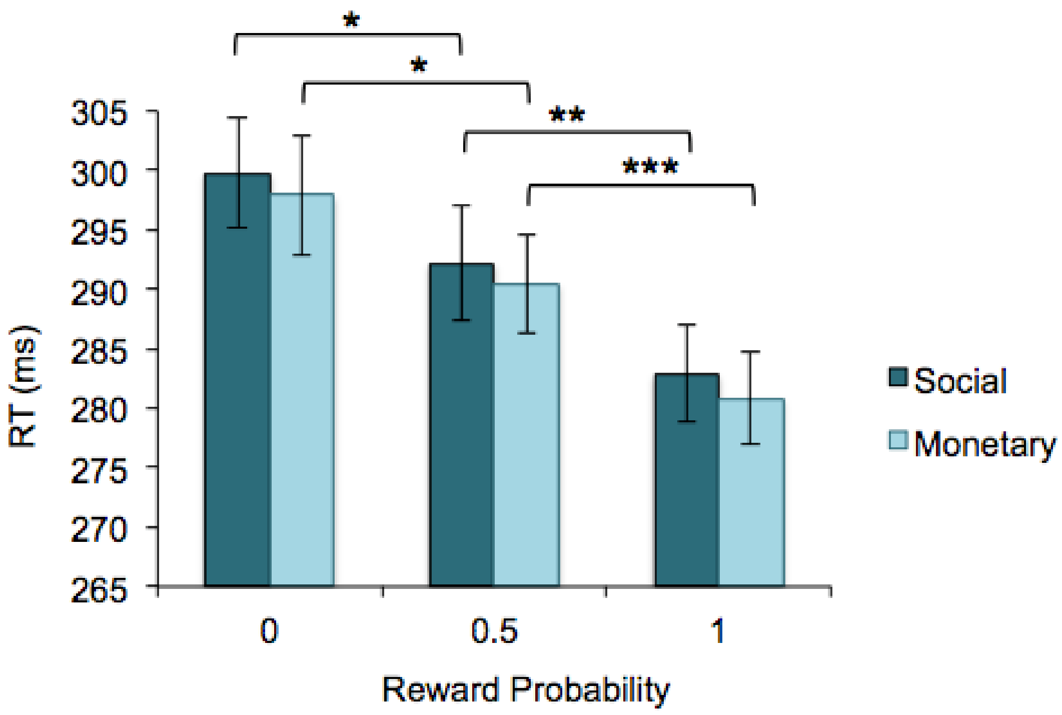

Fig. S1. Mean RT for each probability level on the social and monetary reward tasks (*M*±*SE*).
*** $p < .001$ ** $p < .01$, * $p < .05$.

SM.2 Validation of Experimental Paradigm (2)

The Social Reward Questionnaire for Adolescents (*SRQ-A*; Foulkes et al., 2017) assesses individual differences in the value of different aspects of social reward. This self-report questionnaire consists of 20 items, each beginning with 'I enjoy' and then describing a different type of social interaction, each representing a distinct domain of social reward: *Admiration, Negative Social Potency, Passivity, Prosocial Interaction*s, and *Sociability* (**Table S2**). Participants are asked to consider the item in relation to all their social interactions, e.g., friends, colleagues/classmates or people they have just met. Responses are given on a 1 to 7 scale (1=Disagree strongly, 7=Agree strongly). Each subscale has good psychometric properties and has been shown as having a unique pattern of associations with external measures, providing support for the meaning of each subscale (Foulkes et al., 2017).

Table S2. Detail of *SRQ-A* subscales.

***SRQ-A* subscale**	**Description**	**Example item**
Admiration	Being flattered, liked and gaining positive attention	*'I enjoy getting praise from others'*
Negative Social Potency (NSP)	Being cruel, antagonistic and using others for personal gains	*'I enjoy embarrassing others'*
Passivity	Giving others control and allowing them to make decisions	*'I enjoy following someone else's rules'*
Prosocial Interactions	Having kind, reciprocal relations	*'I enjoy treating others fairly'*
Sociability	Engaging in group interactions	*'I enjoy going to parties'*

Participants' *SRQ-A* scores were used to run a second validation check, in which the relationship between mean RT and participants' self-reported enjoyment of different types of social rewards (*SRQ-A* subscales) was examined using correlational analyses. The purpose of this analysis was to determine whether participants were differentiating between the two reward domains (social vs. monetary), as opposed to simply being influenced by the point gain in a domain-general manner, regardless of the specific nature of the reward symbol (Demurie et al., 2012).

Given that the Facebook like symbol represents social admiration/approval it was hypothesised that participants reporting greater enjoyment of admiration (*SRQ-A Admiration* score) would show faster response times to the social rewards, but not the monetary rewards, used in the experimental task. To assess the degree to which any association between social reward task performance and *SRQ-A Admiration* scores was specific to enjoyment of admiration, as opposed to a more general enjoyment of social rewards, scores on the other four *SRQ-A* subscales were included as exploratory variables. Benjamini and Hochberg False Discovery Rate (Benjamini & Hochberg, 1995) was used to control for the probability of making a Type 1 error on multiple comparisons (**Table S3**).

Table S3. Correlations between reward task performance (mean RT) and *SRQ-A* subscales.

		SRQ-A				
**Reward**	**Probability**	***Admiration***	***NSP***	***Passivity***	***Prosocial***	***Sociability***
**Social**	0	**-.287****	.032	-.188	-.099	-.037
	0.5	**-.377****	.112	-.110	-.120	-.119
	1	**-.287***	.077	-.068	-.061	-.104
**Monetary**	0	-.117	.027	-.067	-.059	-.078
	0.5	-.196	-.022	-.049	.000	-.113
	1	-.208	-.035	-.056	.057	-.079

Corrected *p* values are shown. *N* = 80. ** $p < .01$, * $p < .05$.

SM.3 Non-verbal ability

The matrix reasoning subscale of the WASI (Wechsler, 1999) was originally intended to be used as an age-standardised assessment of non-verbal ability. However, contrary to evidence that relational reasoning continues to improve in late childhood and throughout adolescence (Crone 2009; Dumontheil, 2014; Dumontheil et al., 2010), T-score conversion of raw scores according to the WASI manual serves to decrease the scores of younger participants and increase those of older participants which provoked concerns regarding the suitability of this measure as an age-standardised assessment of non-verbal ability. Both age-standardised and raw scores on the matrix reasoning subscale were significantly correlated with participant age (T-scores: r (80) = .422, p > .001; raw scores: (r (80) = .265, p = .017). However, WASI scores were not significantly correlated with task performance, liking of the reward symbols, or social anxiety symptoms. Thus, due to our concerns regarding the suitability of this measure as a standardised assessment of non-verbal ability it was not included as a predictor in developmental models, as there were no a priori hypotheses regarding the relationship between reward processing and relational reasoning.

SM.4 Age effects on anxiety measures

Hierarchical linear regression models were used to assess the relationship between self-reported social anxiety symptoms (*LSAS* subscales; Liebowitz, 1987), general trait anxiety (*STAI;* Spielberger, 1973) and age. For each anxiety measure, linear (*Step 1*) and quadratic (*Step 2*) age regressors were added in turn, and improvements in model fit at each step were assessed by examining the significance of the *F* change. Based on

epidemiological findings that SAD onset rates of onset peak in early adolescence (Beesdo et al., 2010; Stein, 2006; Kessler et al., 2005), it was predicted that SAD symptoms would likely either decrease or remain stable with age.

Age was a significant negative linear predictor of *LSAS Social Interactions*, accounting for 5.9% of the variance in scores (β = -.244, p = .029), but did not significantly predict *LSAS Performance* (β = -.157, p = .165). Age was also a significant negative linear predictor of *STAI*, accounting for 5.5% of variance in scores (β = -.235, p = .036). Inclusion of a quadratic age regressor did not improve model fit for any measure (**Table S4**; p's >.497).

Table S4. Effects of age on self-reported anxiety scores.

	**LSAS Social Interactions**				**LSAS Performance**				**STAI**			
	R^2	$F\Delta$	$pF\Delta$	β	R^2	$F\Delta$	$pF\Delta$	B	R^2	$F\Delta$	$pF\Delta$	β
Step 1	**.059**	**4.93**	**.029**		.025	1.97	.165		**.055**	**4.56**	**.036**	
Age				**-.244***				-.157				**-.235***
Step 2	.065	0.47	.497		.026	0.09	.764		.058	0.20	.659	
Age				-.852				-.431				-.632
Age^2				.613				.276				.400

Summary of hierarchical regressions investigating linear (age) and quadratic (age^2) effects of age on self-reported assessments of social anxiety symptoms (*LSAS* subscales), general trait anxiety (*STAI*). N = 80. * p < .05.

SM.5 Exploratory post hoc analysis: Uncertainty

Based on visual examination of the data in **Fig 3**, exploratory post hoc analyses were conducted to examine the possibility that responses to uncertain (P = 0.5) social rewards changed with age. Separate repeated measures ANOVAs were conducted for each reward task, with reward probability (0, 0.5, 1) as a within subjects factor and subjective liking rating of the relevant reward symbol included as covariate. A median split was used to group participants by age (< 20.5 years, > 20.5 years), and this was included as a between subjects factor. Pairwise comparisons (Bonferroni corrected) were consistent with visual examination of the social reward task data (**Table S5; Fig. S2**).

Table S5. Mean RTs across probability levels for younger (< 20.5 years) and older (> 20.5 years) participants on the social and monetary reward tasks.

		Pairwise Comparisons: *A* - *B* (SE)	
**Reward Task**	**Probability (*A* - *B*)**	**< 20.5 years**	**> 20.5 years**
Social	0 – 0.5	**11.28 (3.78) ***	3.86 (3.78)
	0 – 1	**16.91 (3.66) *****	**16.92 (3.66) *****
	0.5 – 1	5.62 (23.74)	**13.06 (3.74)****
Monetary	0 – 0.5	7.96 (3.53)	6.87 (3.53)
	0 – 1	**18.34 (4.00) *****	**15.81 (4.00)****
	0.5 – 1	**10.38 (2.59) *****	**8.94 (2.59)****

Participants were split at the median age (20.5 years). Corrected *p* values are shown.
*** *p* < .001, ** *p* < .01, * *p* < .05.

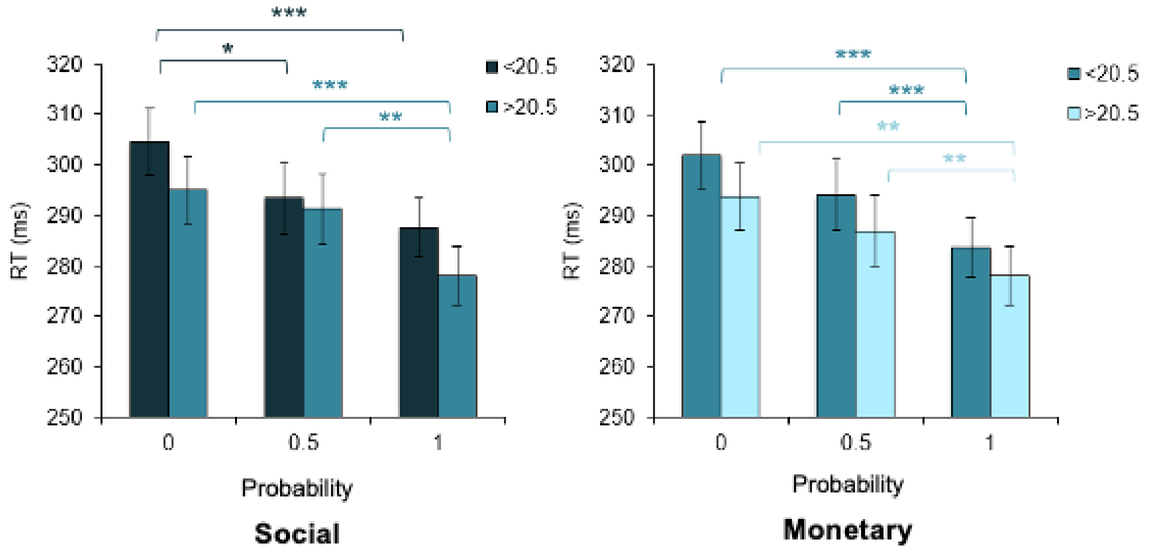

Fig. S2. Mean RTs across probability levels for younger (< 20.5 years) and older (> 20.5 years) participants on the social and monetary reward tasks. Participants were split at the median age (20.5 years). Corrected *p* values are shown. *** *p* < .001, ** *p* < .01, * *p* < .05.

SM.6 Effects of social anxiety on subjective liking ratings of the reward symbols.

The association between participants' self-reported anxiety symptoms and subjective liking ratings of the social and monetary reward stimuli was assessed using hierarchical linear regression, first controlling for symbol familiarity (*Step 1*). *STAI* (*Step 2*) and the two *LSAS* subscales (*Step 3*) were then added as predictors in turn and improvements in model fit at each step were assessed by examining the significance of the *F* change (**Table S6**).

Table S6. Effects of social anxiety on subjective liking ratings of the reward symbols.

	Symbol Liking							
	Facebook Like				£ Symbol			
	R^2	$F\Delta$	$pF\Delta$	β	R^2	$F\Delta$	$pF\Delta$	β
Step 1	**.172**	**16.20**	**< .001**		**.185**	**17.67**	**< .001**	
Familiarity				**.415*****				**.430*****
Step 2	.174	0.22	.640		.185	0.03	.862	
Familiarity				**.406*****				**.426*****
STAI				-.049				.056
Step 3	.188	0.65	.523		.193	0.39	.681	
Familiarity				**.392****				**.424*****
STAI				-.061				-.048
LSAS-S				-.196				-.088
LSAS-P				.234				.160

Summary of hierarchical regressions investigating the relationship between social anxiety (LSAS *Social Interactions* and *Performance* subscales) on subjective liking ratings of the social and monetary reward symbols. Symbol familiarity was controlled for as the first step of the model, followed by trait anxiety (*STAI*). *N* = 80. LSAS-*S: LSAS Social Interactions;* LSAS-*P: LSAS Performance*; ** $p < .01$, *** $p < .001$.

Supplementary References

Beesdo, K., Pine, D. S., Lieb, R. & Wittchen, H.-U. Incidence and Risk Patterns of Anxiety and Depressive Disorders and Categorization of Generalized Anxiety Disorder. *Arch. Gen. Psychiatry* **67**, 47 (2010).

Benjamini, Y., & Hochberg, Y. (1995). Controlling the False Discovery Rate: A Practical and Powerful Approach to Multiple Testing. *Journal of the Royal Statistical Society. Series B (Methodological)*, *57*, 289–300.

Crone, E. A. (2009). Neurocognitive development of relational reasoning. *Developmental Science*, *12*(1), 55–66.

Demurie, E., Roeyers, H., Baeyens, D. & Sonuga-Barke, E. The effects of monetary and social rewards on task performance in children and adolescents: Liking is not enough. *Int. J. Methods Psychiatr. Res.* **21**, 301–310 (2012).

Dumontheil, I. (2014). Development of abstract thinking during childhood and adolescence: The role of rostrolateral prefrontal cortex. *Developmental Cognitive Neuroscience*, *10*, 57–76.

Dumontheil, I., Houlton, R., Christoff, K., & Blakemore, S.-J. (2010). Development of relational reasoning during adolescence. *Developmental Science*, *13*(6), F15–F24.

Foulkes, L., McCrory, E. J., Neumann, C. S. & Viding, E. Inverted Social Reward: Associations between Psychopathic Traits and Self-Report and Experimental Measures of Social Reward. *PLoS One* **9**, e106000 (2014).

Foulkes, L., Neumann, C. S., Roberts, R., McCrory, E., & Viding, E. (2017). Social Reward Questionnaire—Adolescent Version and its association with callous–unemotional traits. *Royal Society Open Science*, *4*(4), 160991.

Foulkes, L., Viding, E., McCrory, E. & Neumann, C. S. Social Reward Questionnaire (SRQ): development and validation. *Front. Psychol.* **5**, 1–8 (2014).

Kessler, R. C. *et al.* Lifetime Prevalence and Age-of-Onset Distributions of DSM-IV Disorders in the National Comorbidity Survey Replication. *Arch. Gen. Psychiatry* **62**, 593 (2005).

Liebowitz, M. R. Social phobia. *Mod. Probl. Pharmacopsychiatry* **22**, 141–73 (1987).

Rademacher, L. *et al.* Dissociation of neural networks for anticipation and consumption of monetary and social rewards. *Neuroimage* **49**, 3276–85 (2010).

Spielberger, C. D. *Manual for the State-Trait Anxiety Inventory (STAI)*. (Consulting Psychologists Press, 1973).

Spreckelmeyer, K. N. *et al.* Anticipation of monetary and social reward differently activates mesolimbic brain structures in men and women. *Soc. Cogn. Affect. Neurosci.* **4**, 158–65 (2009).

Stein, M. B. An epidemiologic perspective on social anxiety disorder. *J. Clin. Psychiatry* **67** Suppl 1, 3–8 (2006).

Wechsler, D. *Wechsler Abbreviated Scale of Intelligence (WASI)*. (Harcourt Asessment, 1999).
